# Elective Thoracic Surgical Resections for Pulmonary Arteriovenous Malformations ‐ A 16 Year Single‐Center Experience

**DOI:** 10.1002/pul2.70037

**Published:** 2025-01-07

**Authors:** May Al‐Sahaf, Jon Anderson, Jayanta Nandi, Ali Alsafi, Claire L. Shovlin

**Affiliations:** ^1^ Department of Cardiothoracic Surgery Hammersmith Hospital, Imperial College Healthcare NHS Trust London UK; ^2^ National Heart and Lung Institute, Imperial College London UK; ^3^ Department of Imaging Imperial College Healthcare NHS Trust London UK; ^4^ Respiratory Medicine Imperial College Healthcare NHS Trust London UK

**Keywords:** asymptomatic, hereditary hemorrhagic telangiectasia, hypoxemia, ischemic stroke, right‐to‐left shunt

## Abstract

Pulmonary arteriovenous malformations (PAVMs) cause cerebral abscess and ischemic stroke due to paradoxical emboli, risks that are increasingly recognized. We report the evolving placement of thoracic surgery in multi‐disciplinary team management of PAVMs that were sporadic or associated with hereditary hemorrhagic telangiectasia. From 1983 to 2006, all patients receiving elective treatment had embolization. Between January 2006 and July 2022, 24 of 714 (3%) patients reviewed at our institution underwent elective surgical resection of one or more PAVMs. Initially, the bar for elective surgery had been set very high, and only patients with persistent symptoms of cerebral ischemia after maximal embolization and medical therapy were referred. As PAVM natural history, follow‐up radiation exposures for residual PAVMs, and good surgical outcomes were appreciated, PAVM resection became part of the discussion for highly localized, very complex PAVMs which we consider are best treated surgically. Elective surgical intervention may be considered for other selected patients.

## 1 Case Description

Between 2006 and 2022, we reviewed 714 patients with pulmonary arteriovenous malformations (PAVMs). Intervention was undertaken in 555 (78%) while 159 (22%) were managed conservatively, usually due to small size of PAVMs. Most patients (531/555, 96%) were treated by embolization. 527 were treated electively, with 4 requiring emergency treatment for hemoptysis/hemothorax. Twenty‐six patients were treated surgically‐ two as emergencies for massive hemoptysis, and 24 who are the focus of this report.

**Figure 1:**
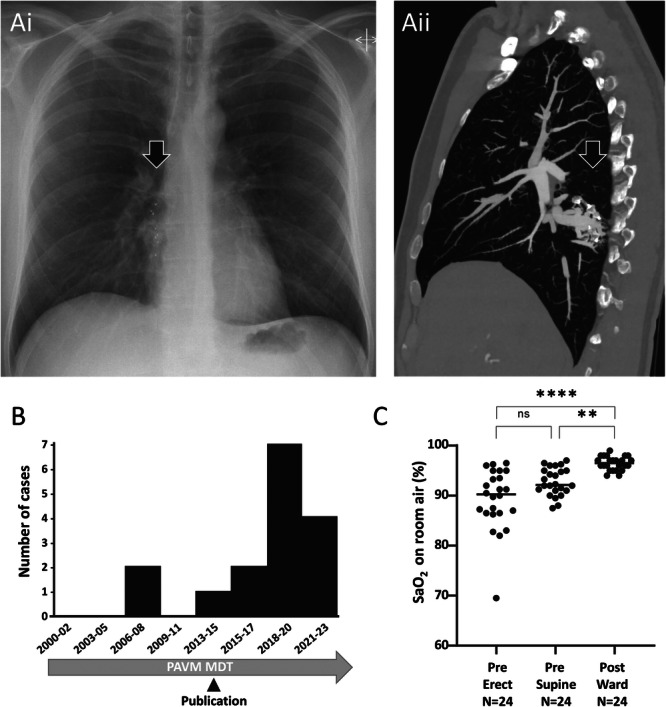
(A) Complex right lower lobe PAVM following previous embolization with several Amplatzer vascular plugs. Preoperative images. (i) Chest x‐ray indicating Amplatzer plug radiopaque tips and additional soft tissue shadowing (arrowed). (ii) Sagittal images from a contrast enhanced thoracic CT scan demonstrating a persistent venous sac with multiple small residual feeding arteries, the diameter and position of which preclude complete embolization. (B) Case series: Serial number of elective thoracic surgical cases, noting date overseas audit of cumulative radiation doses in patients with PAVMs was published [4]. Cases from the third series (2023‐) are the subject of separate Case Reports with specific educational/training foci. (C) Pre and postoperative oxygen saturation (SaO_2_). SaO_2_ is a validated biomarker of right‐to‐left shunting [5], and did not differ between Series 1 and Series 2 pretreatment (means 89.2% *vs.* 89.6%). *p* values were calculated by Dunn's test post Friedman (**p < 0.01, ****p < 0.001).

The 24 patients receiving elective surgery were aged 17–80 years (mean 39 years), 11 (46%) were male, and 17 had confirmed hereditary hemorrhagic telangiectasia (HHT) spanning *ACVRL1*, *ENG* and *SMAD4* genotypes [1, 2, 3]. Ten (42%) had already undergone maximal embolization of their PAVMs: further embolization was not feasible due to feeding arterial diameters too small/too numerous for further embolization (Figure 1A) but they had ongoing severe hypoxemia (*N* = 3), neurological symptoms (*N* = 3), hemoptysis (*N* = 3), or severe pain following pulmonary infarction from proximal embolization at another institution. Fourteen patients with no previous embolization had very complex PAVMs with innumerable small feeding arteries, where embolization was thought unlikely to obliterate right‐to‐left shunting completely. In these cases, surgical resection was also offered to the patients, and following a joint process of decision making, decided to be the most suitable first‐line treatment.

The surgical aim was to remove all abnormal tissue, as remnant thin‐walled AV fistulous tissue would risk local recurrence. The extent of resection was, therefore, decided based on accessibility, control and extent of abnormal tissue and is described in detail in the Data Supplement. 11/24 (46%) patients were able to undergo a limited lung sparing procedure (wedge or segmentectomy). There was no operative mortality and no intra‐operative complications. Median lengths of stay were < 1 day (range 0–1.5 days) in the surgical intensive care unit, and 4 days (range 2–12 days) in hospital. All patients were discharged home, no patient required to be discharged with a tube thoracostomy/Heimlich valve or required rehabilitation/nursing home care post operatively, and the 30‐day readmission rate was zero. The mean postoperative follow‐up was 10.3 months (range 1.0–150 months). All surgical and incisional related pain resolved by 6 months, although one patient was reporting issues considered possibly related to scar tissue at 28 month follow up. SaO_2_ improved significantly post‐surgery (Figure 1C). Eight patients reported markedly improved exercise tolerance, none have had further hemoptysis, and all except one reported no further neurological symptoms.

Earlier at our institution (1983–2006), all patients receiving elective treatment for PAVMs had embolization. As discussed below, greater experience that significant new PAVMs were unlikely in adult‐life, was accompanied by recognition of the radiation burden involved in following up patients with residual PAVMs to assess for enlargement and re‐embolization. Potential resection became part of the management discussion for patients with highly localized, very complex PAVMs, and following commencement of elective surgery in 2006, the overall ratio of surgery:embolization at our institution has been 1:22 (24:531).

## 2 Discussion

Our experience led us to recognize that there are subgroups of patients in whom elective surgery could be offered. Here we report these elective surgical resections, performed where expert embolization was also available as a treatment option.

PAVMs provide aberrant communications between pulmonary arteries and veins, resulting in a fraction of the right ventricular output bypassing normal pulmonary capillaries [5]. As detailed by the British Thoracic Society Clinical Statement, despite impaired gas exchange, most patients are asymptomatic; hypoxic pulmonary hypertension does not develop because patients have hypoxemia without alveolar hypoxia; chest pain is not a feature of untreated PAVMs; and hemorrhage from PAVMs is rare outside of pregnancy. However, treatment of PAVMs is recommended irrespective of symptoms, primarily to reduce the risk from paradoxical emboli [1, 2, 3, 5, 6, 7, 8, 9]. The rate of brain abscess (which requires neurosurgical drainage) was 6.2% (27/435) in one series after correction for ascertainment bias [6]. In this series, 13.5% (5/37) of abscess‐affected patients had PAVMs where all feeding arteries were ≤ 3 mm in diameter, and a modest reduction in right‐to‐left shunting (corresponding to a 1% increase in SaO_2_) translated to a 10.5% risk‐reduction [6]. As summarized recently, ischemic stroke rates have exceeded 10% in all PAVM series, with silent brain infarction at least twice as frequent [7]. The 2005–2014 United States Nationwide Inpatient Sample database showed ischemic stroke occurred a decade earlier with PAVMs than routine stroke, with patients losing 9 extra healthy‐life‐years [8]. Further, primary and secondary prevention with antiplatelet agents can be more nuanced since most PAVMs occur in association with HHT [10], when there is often reluctance of patients to take antiplatelet therapy, despite expert consensus confirming antiplatelet agents can be used [1, 2]. For all of these reasons, maximal reduction of right‐to‐left shunting is important.

Before the 1980s, surgical resection was the only available treatment for PAVMs, and they were usually managed conservatively. Following the development of percutaneous transcatheter embolization, this became first‐line treatment for most PAVMs, with high levels of technical success and low rates of complications in experienced centers [5]. For instance, our institution has reported pre‐ and post‐embolization data for 712 patients, demonstrating improved oxygenation, reduced complications, and pulmonary artery pressure responses. However, very complex PAVMs pose a particular challenge to endovascular treatment as some have innumerable small feeding vessels, which may preclude complete AVM occlusion. Whilst partial embolization of such lesions may reduce the risk of paradoxical emboli, it often does not eliminate it completely. Furthermore, if proximal embolization of such lesions is undertaken, pulmonary infarction may ensue causing severe pleuritic pain and systemic inflammation, in addition to the risk of reperfusion from pulmonary collaterals or systemic arterial supply that can result in hemoptysis [3, 9].

In conclusion we have shown that in selected cases, surgical resection can be considered outside emergency indications, even for patients with HHT where there was previously caution due to uncertainty whether further PAVMs would develop. Greater experience, appreciation of PAVM natural history and recognition of follow‐up radiation burden [4] has resulted in a lower threshold for considering surgical resection at our center. We show that where resection can be achieved with limited loss of lung parenchyma, surgical resection of PAVMs is associated with low mortality and low postoperative complications. Critically, it can be curative in patients with localized very complex PAVMs, where embolization would leave residual shunting.

## Author Contributions

J.A., M.A.‐S, A.A and C.L.S. ran the PAVM MDT; J.A. M.A.‐S, and J.N. managed surgical cases; A.A. performed embolizations and provided imaging reviews; C.L.S. reviewed patients medically. Manuscript text was written by J.A., A.A. and C.L.S. Data analysis was by C.L.S. Figures were generated by A.A. and C.L.S. C.L.S. is the guarantor for the manuscript. All authors reviewed and approved the final manuscript.

## Ethics Statement

Ethical approvals was from the Hammersmith, Queen Charlotte's and Chelsea and Acton Hospital Research Ethics committee (LREC 2000/5764 “Hammersmith Hospital patients with pulmonary arteriovenous malformations (PAVMs) and hereditary hemorrhagic telangiectasia”).

## Conflicts of Interest

The authors declare no conflicts of interest.

## Supporting information

Supporting information.

## Data Availability

Fully anonymised aggregate data are provided in the Data Supplement. Any further data sharing would be on the basis that it provided no potential to breach patient anonymity.
