# Supplementary material for: Elective Thoracic Surgical Resections for Pulmonary Arteriovenous Malformations ‐ A 16 Year Single‐Center Experience
Source: Pulm Circ. 2025 Jan 7;15(1):e70037. doi: 10.1002/pul2.70037 (PMC11705419; doi:10.1002/pul2.70037)
Supplement: Supplementary file 1 — Supporting information. [file PUL2-15-e70037-s001.docx]

**Elective thoracic surgical resections for pulmonary arteriovenous malformations**

**- a 16 year single-centre experience.**

**May Al-Sahaf*,^1^ Jon Anderson*^1^, Jayanta Nandi^1^, Ali Alsafi^2^, Claire L. Shovlin^3,4,^**

** These authors contributed equally*

^1^ Department of Cardiothoracic Surgery, Hammersmith Hospital, Imperial College Healthcare NHS Trust, London, UK ^2^Department of Imaging, Imperial College Healthcare NHS Trust, London, UK. ^3^National Heart and Lung Institute, Imperial College, London, UK ^4^Respiratory Medicine, Imperial College Healthcare NHS Trust, London, UK

**DATA SUPPLEMENT**

[**Background** 2](#_Toc126528578)

[**Patients and Methods** 2](#_Toc126528579)

[*Study populations* 2](#_Toc126528580)

[*Selection for elective surgery 2006-2022* 2](#_Toc126528581)

[*Current analysis* 3](#_Toc126528582)

[*Perioperative outcomes* 3](#_Toc126528583)

[*Statistical analysis:* 3](#_Toc126528584)

[**Results** 3](#_Toc126528585)

[*Cases and patient population.* 3](#_Toc126528586)

[*Surgical procedures:* 3](#_Toc126528587)

[*Immediate complications:* 4](#_Toc126528588)

[*Post-operative outcomes:* 4](#_Toc126528589)

[*Hospital stays:* 4](#_Toc126528590)

[**Discussion:** 4](#_Toc126528591)

[**Supplementary Tables:** 6](#_Toc126528592)

[*Table S1: Summary of operative interventions in 24 patients* 6](#_Toc126528593)

[*Table S2: Surgical Procedures in 24 patients undergoing PAVM resection* 6](#_Toc126528594)

[*Table S3: Follow up of 24 patients following elective PAVM surgery*  7](#_Toc126528595)

[**References:** 8](#_Toc126528596)

## **Background**

Pulmonary arteriovenous malformations (PAVMs) are vascular anomalies that provide aberrant communications between pulmonary arteries and veins, resulting in a right-to-left shunt.[1,2] PAVMs are estimated to affect 38 per 100,000 people,[3] and usually occur in association with hereditary hemorrhagic telangiectasia (HHT).[4,5] Hypoxemia due to the right-to-left shunt is usually asymptomatic as a result of compensatory mechanisms such as polycythemia and higher cardiac output.[1,2] More importantly, PAVMs of any size can result in paradoxical emboli, leading to neurological sequelae such as ischemic strokes,[6] silent brain infarcts,[7] brain abscesses,[8-10] and wider complications including myocardial infarction,[11] discitis and other deep-seated infections.[8,12] For example, in a series of 37 cases complicated by a brain abscess, in five (13.5%), all PAVM feeding arteries had a diameter ≤3 mm.[8] Additionally, rarely, patients with PAVMs may present acutely with massive hemoptysis or hemothorax, and require emergency treatment.[13]

Treatment of PAVMs is primarily recommended to reduce the risk from paradoxical emboli,[1,2] where a 1% rise in SaO_2_ is associated with a 10.5% (95% CI, 4.18%–16.36%) reduction in risk of brain abscess.[8] In symptomatic patients, additional treatment benefits include improvement in migraines, hemoptysis and dyspnea. Prior to the 1980s, surgical resection was the only available treatment for PAVMs. Early literature details successful surgical responses to ligation, local excision, segmentectomy, lobectomy or pneumonectomy,[14] though more usually conservative management was applied. In most countries, surgical resection is performed in the emergency hemorrhagic setting. For elective resections, the majority of descriptions are isolated case reports from various institutions,[15-18] although in Japan, elective surgical resection is more commonly performed, with one large series reporting 211 surgical cases.[19]

Since the 1980s, advances in interventional radiology have led to successful application of percutaneous transcatheter embolization to treat single and multiple PAVMs.[20] Today, embolization is the first line treatment for most PAVMs, with high levels of technical success and low rates of complications in experienced centres. From our institution, pre and post-embolization data are reported for 712 patients, demonstrating improved oxygenation,[21-24] pulmonary artery pressure responses,[25] and reduced rates of ischemic strokes,[26] and cerebral abscess,[26] with improvements in migraines also reported.[27] However, very complex PAVMs pose a particular challenge to endovascular treatment as some have innumerable small feeding vessels, which may preclude complete AVM occlusion. Whilst partial embolization of such lesions may reduce the risk of paradoxical emboli, it does not eliminate it. Furthermore, if proximal embolization of such lesions is undertaken,

pulmonary infarction may ensue in addition to the development of systemic arterial supply and subsequent haemoptysis.[2,14]

Our experience has led us to recognise that there are subgroups of patients in whom

elective surgery could be offered – in emergency (manuscript in preparation) and elective settings either where PAVMs are localised but very complex precluding complete embolization (main manuscript Figure 1), or potentially where patients spontaneously state a preference for surgery as their primary treatment. The goal of this study was to report the outcomes of these elective surgical resections performed where expert embolization was also available as a treatment option.

## **Patients and Methods**

### ***Study populations***

Elective surgical resections were considered from January 2006, a period when 531 patients were treated by embolization in 659 embolisation sessions. For all PAVM patients, clinical symptoms recorded included those potentially related to PAVMs (migraines, strokes and/or transient ischemic attacks, impaired exercise capacity/dyspnoea/fatigue), together with symptoms such as epistaxis, from underlying HHT. As described elsewhere,[21-26] baseline physiological parameters included oxygen saturations (SaO_2_) which were measured by pulse oximetry in the supine and erect postures for 10 mins, since erect SaO_2_ better reflects right-to-left shunt size than SaO_2_ in other postures, but supine may be more appropriate to compare to ward-based measurements. All patients were advised to use prophylactic antibiotics prior to dental and surgical procedures.[1,10] Patients’ management including evaluation of suitability for conservative management, embolization, or surgery, was discussed in multi-disciplinary team (MDT) meetings.

A separate cohort of patients presented acutely with massive hemoptysis and within this larger group of patients requiring emergency surgery for vascular abnormalities, a small proportion of patients had PAVMs. They were excluded from the current series of elective surgery cases.

### ***Selection for elective surgery 2006-2022***

Consideration for elective surgery was made on a case-by-case basis in a multi-disciplinary manner. From 1999, the same consultants facilitated the development of treatment approaches. The core MDT team was augmented by additional members in time. All PAVM images were reviewed by experienced radiologists. These personnel worked together, combining expertise in defining patients suitable for embolization, primary surgery and surgery following previous embolization.

Primary surgery was considered in patients with a single very complex PAVM, described as those with numerous small feeding vessels: this is because embolizing these lesions may not entirely abrogate right-to-left shunting. Surgery following previous embolization was considered for patients with persistent and/or recurrent PAVMs, which were not amenable to further embolization, and in whom elimination of right-to-left shunting was considered achievable with limited loss of lung parenchyma. This was the case for patients when a complex PAVM had multiple tiny residual feeding vessels or had re-perfused via multiple small pulmonary artery-to-pulmonary artery collaterals, which were too small for embolization.

### ***Current analysis***

Cases referred for surgery were evaluated retrospectively with ethical approvals from the Hammersmith, Queen Charlotte’s and Chelsea and Acton Hospital Research Ethics committee (LREC 2000/5764 “Hammersmith Hospital patients with pulmonary arteriovenous malformations (PAVMs) and hereditary haemorrhagic telangiectasia”). Data were collected from electronic patient records and institutional records; performed as part of the routine investigations and clinical management of patients with PAVMs. In keeping with the Curaçao Criteria,[28] clinical HHT was diagnosed in the presence of at least three criteria; epistaxis, characteristic telangiectasia, visceral involvement and an affected first-degree family member. HHT was also diagnosed by a pathogenic or likely pathogenic variant in a gene known to cause HHT.[4,29] Clinical details at the time of surgery and at the latest follow up in 2022 were evaluated.

### ***Perioperative outcomes***

Operative details recorded included surgical technique (wedge resection, lingulectomy, segmentectomy, and lobectomy), lung zone operated on (upper, middle, or lower) as well as surgical approach (thoracotomy vs video-assisted thoracoscopic surgery [VATS]). Patients who had a complication or died during the 30-day post-operative period were considered to have experienced a composite complication or peri-procedural mortality, respectively. Post-operative complications recorded spanned all pulmonary complications such as pneumothorax, atelectasis, prolonged air leak as well as post-operative wound infection, pain requiring input from specialist pain team or psychological distress requiring specialist mental health team input. Total post-operative stay in the cardiothoracic intensive care unit and hospital stay was obtained. Pre-operative oxygen saturations (including average supine and erect saturations averaged over 4 minutes after 7-10 minutes in the relevant posture), were compared to saturations at discharge from hospital following surgery.

### ***Statistical analysis:***

Descriptive statistics were generated using GraphPad Prism 9.0.0 (Graph Pad Software Inc, San Diego). Two group comparisons of unpaired data were performed using the Mann Whitney test. Three group comparisons of paired data were performed using the Friedman test with Dunn’s test to derive pairwise comparisons. Categorical variables were compared between patients using Fisher’s exact test.

## **Results**

### ***Cases and patient population:***

Between January 2006 and June 2022, 714 patients with PAVMs were reviewed at Hammersmith Hospital, with 159 (22%) managed conservatively, usually due to small size of PAVMs, and 555 (78%) receiving intervention.

Of the 555 treated, 531 (96%) were treated by embolization in a total of 659 embolization sessions. Four of 659 embolization sessions were for emergencies following transfer to our institution as a regional referral centre. All 4 were female (3 pregnant), and all received treatment during the emergency admission for hemoptysis (N=3) or hemothorax (N=1): All hemorrhage settled after embolization and no re-intervention was required.

Of the 555 treated, 26 were treated surgically - two (both males), in emergency procedures for massive hemoptysis and 24 who underwent elective thoracic surgical resection and are the subject of the current manuscript.

The mean age of the 24 patients was 39 years, ranging from 17 to 80 years at the time of surgery. At least 17 of the 24 had underlying HHT spanning the 3 major genotypes (*ENG*, *ACVRL1*, and *SMAD4*), with one a mosaic. None of the patients had cyanotic congenital heart disease or single ventricle physiology, which is important since patients with heterotaxy or interrupted vena cava post Glenn or Fontan procedures are known to develop pulmonary AVMs.

Oxygen saturations (SaO_2_) prior to surgery ranged from 69.5-96.5% (mean 89%) in the erect posture.

At the time of consideration for lung resection, 10/24 (42%) had already undergone maximal embolization of their PAVMs, with a median of 2 (range 0 to 6) embolization procedures. Of this cohort of 10, three had persistent profound hypoxemia, three had neurological complications, 3 had experienced hemoptysis (2 planning future pregnancies), and one had severe pain following pulmonary infarction from proximal embolization at another institution. The remaining cohort with no previous embolization had very complex PAVMs with innumerable small feeding arteries, where embolization was thought unlikely to obliterate right-to-left shunting completely. In these cases, surgical resection was also offered to the patients, and following a joint process of decision making, decided to be the most suitable first line treatment. Of this cohort of 14, five had profound hypoxemia, and 5 were symptomatic (from hypoxemia, syndrome of inappropriate antidiuretic hormone secretion (SIADH) or neurological complications).

###

### ***Surgical procedures:***

The number of PAVMs affecting the right and left lungs were similar (13 vs 11). (Table S1) Post 2018, procedures were planned for video assisted thoracoscopic resections (VATS) though consented for conversion to thoracotomy which was required in 8 cases.

All AVMs were in the lung parenchyma. The lesions were identifiable as pulsatile masses comprising a mesh of thin-walled vessels risking easy uncontrolled bleeding. Surgical principles involved approaching the more normal proximal vessels with careful surgical dissection to control and ligate the inflow vessels. The main feeding vessel to the AVM was identified on the CT reconstruction and intraoperatively clamped. The AVM itself was easy to identify when the lung was collapsed as it stayed perfused and prominent. When the feeding vessel was clamped intra-operatively, the saturations usually rose and the vessel was then ligated.

The aim was to remove all abnormal tissue as remnant thin-walled AV fistulous tissue would risk local recurrence. The extent of resection was, therefore, decided based on accessibility, control and extent of abnormal tissue. A generous wedge or formal segmentectomy was then undertaken to encompass all the likely affected lung parenchyma. In total, 11/24 (46%) patients were able to undergo a limited lung sparing procedure (wedge or segmentectomy, Table S2)

All patients were recommended to have prophylactic anticoagulation based on latest HHT recommendations, but not all were agreeable to this. All histopathology was consistent with pulmonary arteriovenous malformation with no evidence of malignancy.

### ***Immediate complications:***

There was no operative mortality and no intra-operative complications. Surgical intensive care unit stay ranged from 0 to 1.5 days (median <1 day).

### ***Post-operative outcomes:***

There was no re-intervention in any patient. 30-day composite complications occurred in 11/24 patients undergoing elective surgical PAVM resection. 6/24 (25%) patients developed pneumothoraces after chest drain removal, all resolving with no long-term consequences. Four patients developed prolonged air leakage, 3/24 patients had atelectasis, 3/16 patients experienced significant post-operative pain requiring pain services, with 2/24 patients experiencing opioid sensitivity complications. Of the 24 patients, 7 developed an infection (respiratory, wound or urinary) responding to oral antibiotics. An early patient experienced exacerbation of longstanding psychological issues, leading to additional pain team consultations and

TENS provision for a subsequent patient deemed at possible risk due to complex social, psychiatric and medication interactions.

### ***Hospital stays:***

Overall hospital stay ranged from 2-12 (median 4) days. The longest hospital admission was 12 days in a female who developed a pneumothorax, following an elective left upper lobectomy, which was monitored for an extra 48 hours on suction. All patients were discharged home, and no patient required to be discharged with a tube thoracostomy/Heimlich valve or required rehabilitation/nursing home care post operatively. The 30-day readmission rate was zero.

***Follow up:***

The mean post-operative follow-up was 10.3 months (range 1.0–150 months). Table S3 summarises clinical and physiological follow up data. All surgical and incisional related pain, in both cohorts resolved by 6 months, although one patient was reporting issues considered possibly related to scar tissue at 28 month follow up.

At the time of the most recent or final assessment, no patient had developed hemoptysis, no patient had developed a brain abscess, and antibiotic prophylaxis recommendations were stopped for all where complete resection of all PAVMs had been achieved. No patient had developed an ischemic stroke, although one patient with pre-operative recurrent ischemic strokes/TIAs despite maximal embolization, suffered further symptoms (gradual left leg weakness lasting a few days) 1 month following surgery. The gradual onset of the weakness was not considered fully compatible with a clinical stroke, but the patient was managed with enhanced ischemic stroke prophylaxis (antiplatelet agents plus a statin), in addition to broader medical management of HHT.

All patients maintained SaO_2_ (erect) above 94%. Eight reported markedly improved exercise tolerance. Three patients reported particularly marked improvement in exercise capacity, using words such as "supersonic". One whose SaO_2_ increased from 87% to 98%, within 10 months of surgery increased her Veterans Specific Activity Questionnaire (VSAQ) Score from 5 to 9,[30] running 10 kilometres. One patient had a prior 3 year history of presumed SIADH, reporting headaches and impaired cognition at times of hyponatremia that had responded to 1.5L fluid restriction: This was of unknown etiology but when followed up 9 months post-surgery, she had been able to cease fluid restrictions with no recurrence of symptoms and no alternate changes in therapeutics were identified that might have been associated with the clinical improvement.

## **Discussion:**

We have shown in 24 highly selected elective cases that surgical resection of PAVMs can be achieved with no mortality, and good peri-operative and medium-term outcomes, without the need for long-term imaging follow up. This surgery can be offered safely and is effective in improving oxygen saturations as well as

reducing the burden of PAVM-related symptoms and complications both in the short and medium-term. This study provides evidence to help guide the management of patients where PAVMs are localised but very complex precluding complete embolization, and where patients prefer surgery as their primary treatment.

The strengths of our study were the vast experience in PAVM management to enable case selection for surgical resection. Study limitations include its retrospective nature and small sample size, but nonetheless it represents a large modern case series of PAVMs managed surgically at an institution offering longstanding expert embolization with proven outcome improvements post embolization.

Surgery is a common modality in Japan where 211/996 (21%) of patients with PAVMs received surgical resection country-wide with fewer reinterventions than embolization, but a significantly higher proportion of composite complications and longer postoperative hospital stay.[18] A growing number of single case reports suggest curative benefits of surgical resection.[14-17] Through our case series, we demonstrate the types of cases where surgery is preferred over embolization: For patients in whom right-to-left shunting cannot be completely abolished by embolization, but where limited surgical resection offers the possibility of a cure, in our opinion, an informed discussion of a surgical option, recognising the transient but extra side effects from surgery is appropriate. This is particularly for patients whose PAVMs are not amenable to further embolization, but either have persistent neurological symptoms from this stroke-precipitating pathology [6], or are young women planning future pregnancies where hemoptysis[31] or PAVM-induced activity limitation[30] has already occurred. Additionally, a significant proportion of reported patients undergoing surgery had ongoing symptoms or complications despite previous multiple embolization of their PAVMs. The number of embolizations that some patients receive reflects the multiplicity of their PAVMs as opposed to recurrent attempts to treat the same PAVMs. Nevertheless, follow up to ensure resolution of symptoms post embolization is important: The interventional radiologists’ experience of in our study has allowed for appropriate identification of previously treated PAVMs that may not be amendable to complete occlusion or to further embolization therapy.

There remain few recent reports on the surgical resection as first line in Europe and the US. Outcomes in our study shed light on elective surgical resection as a first line treatment. In the presence of an isolated complex PAVM, surgical resection has potential curative benefits that may not be achieved by embolization. In our study, 46% of resections were lung-conserving in nature. Previous retrospective studies on surgically treated PAVM cases reported lower rates of parenchymal-sparing
techniques. Recently video assisted thoracoscopy (VATs) has been employed in the resection of a small PAVMs: 58% of our cases were via VATS, with similar utilisation rates in other institutions.[14,17] Surgical intervention for PAVMs carries similar risks to other thoracic operations- and selection of surgery needs to be in a setting where these risks are outweighed by benefits greater than could be conveyed by embolization. Historically, perioperative mortality ranged from 0–9%. Patients with underlying co-morbidities should be appropriately screened prior to surgery, for suitability for anesthesia and single lung ventilation, and because removal of the low resistance shunt risks precipitating acute increases in pulmonary arterial pressure (PAP). This was discussed,[32] following pre/post embolization PAP measurements in 98 patients.[25] Notably, at least two patients with pulmonary arterial hypertension have died of acute heart failure within 12 hours of PAVM resection.[33,34]

Pain management is important for patients to comply with chest physiotherapy and ambulation, and to reduce the burden of further post-operative pulmonary complications (PPCs) including, pneumonia and prolonged air leak.[35,36] In this case series, we observed one pneumonia, one parapneumonic collection, and radiologically reported pneumothoraces in 6/24 (25%) cases. All pneumothoraces resolved with conservative measures and hence deemed to be related to chest drain removal technique in smaller lung resection cases (wedge or segmentectomies) and due to areas of residual lung that had not yet expanded in cases with larger volume lung resections (lobectomies). The nature of post-operative complications reported are in keeping with well-established complications following thoracic surgery; the incidence in the current series reflects the highly selected cohort of patients with pre-existing morbidities, and stringent criteria for detection of small pneumothoraces following chest drain removal due to institutional radiological screening protocols. Risk factors for PPCs following pulmonary resection have been identified as age ≥75 years, chronic obstructive pulmonary disease (COPD), body mass index (BMI) ≥30 kg/m^2^, American Society of Anesthesiology (ASA) score ≥3, and current smoking history, of which the latter can be mitigated with appropriate pre-operative work up and optimisation.[36,37]

Early in our series we observed that patients who already had a background of depression or anxiety, could go on to experience significant post-operative pain and psychological distress.[37,38] Since our adoption of preoperative enhanced pain team support for identified at risk individuals, we have not seen further complications of this nature. Provision of TENS machines to enhance patient confidence, careful weaning of analgesics (especially opioids) and enhanced psychosocial support in the perioperative period all help reduce the burden of such complications. We speculate that the increased utilisation of VATS amongst surgeons will contribute to the reduction of post-operative pain from reduced surgical incision size and non-rib-spreading approaches.

## **Supplementary Tables:**

| **Table S1: Operative Interventions in 24 patients** | |
| --- | --- |
| **Resection Type, n (%)**  **Wedge**  **Segmentectomy**  **Lobectomy** | **5 (21)**  **6 (25)**  **13 (54)** |
| **Access, n (%)**  **Thoracotomy**  **VATS** | **10 (42)**  **14 (58)** |
| **Lung Zone, n (%)**  **Upper**  **Middle**  **Lower** | **8 (33)**  **6 (25)**  **10 (42)** |
| **Lung side, n (%)**  **Right**  **Left** | **13 (54)**  **11 (46)** |

### *Table S1: Summary of operative interventions in 24 patient. N, number.*

| **Table S2: Surgical Approach in 24 patients** | | | | | | |
| --- | --- | --- | --- | --- | --- | --- |
|  | **Right** | | | **Left** | | |
|  | **W** | **S** | **L** | **S** | **L** |  |
| **Upper lobe** | 1 | 1 | 1 | 7 | 1 | **11** |
| **Middle Lobe** |  |  | 2 |  |  | **2** |
| **Lower Lobe** |  | 2 | 6 | 1 | 2 | **11** |
|  | **1** | **3** | **9** | **8** | **3** | **24** |

### *Table S2: Surgical Procedures in 24 patients undergoing PAVM resection*

L: Lobectomy, S: Segmentectomy, W: Wedge resection.

|  | | |  |  |
| --- | --- | --- | --- | --- |
|  | | | | |
| **Table S3: Follow up of 24 patients following elective PAVM surgery** | | | | |
|  | **All**  ***N = 24*** | **No previous embolization**  ***n = 14*** | | **Previous embolization**  ***n= 10*** |
| **Length of follow up, months,**  **median (IQR)** | 2.2 (1–150) | 3.7 (1 – 10) | | 13.8 (1 – 149) |
| **Hypoxemic, n (%)** | 0 (0) | 0 (0) | | 0 (0) |
| **PAVM Complications, n (%)**  Ischemic stroke/TIAs  Cerebral abscess  Venous thromboembolism  Syndrome of inappropriate ADH secretion  **Symptoms, n (%)**  Hemoptysis  Dyspnea | 1 (6)  0 (0)  0 (0)  0 (0)    0 (0)  0 (0) | 0 (0)  0 (0)  0 (0)  0 (0)  0 (0)  0 (0) | | 1 (14)  0 (0)  0 (0)  0 (0)  0 (0)  0 (0) |
| **Post-surgical pain, n (%)**  Long term pain at 6 months | 1 (6) | 1 (10) | | 1 (7)* |
| **Mortality, n (%)** | 0 (0) | 0 (0) | | 0 (0) |

### *Table 3: Follow up of 24 patients following elective PAVM surgery*

** Had not reached 6 months but pain pre-procedure post previous embolization. N/n, number. IQR, interquartile range.*

## **References:**

1 Shovlin CL, Condliffe R, Donaldson JW, et al. British Thoracic Society Clinical Statement on Pulmonary Arteriovenous Malformations. *Thorax* 2017 Dec;72(12):1154-1163.

2 Shovlin CL. Pulmonary arteriovenous malformations. *Am J Respir Crit Care Med*. 2014;190(11):1217-1228

3 Nakayama M, Nawa T, Chonan T, et al. Prevalence of pulmonary arteriovenous malformations as estimated by low-dose thoracic CT screening. *Intern Med*. 2012;51(13):1677-81

4 Anderson E, Sharma L, Alsafi A, et al. Pulmonary arteriovenous malformations may be the only clinical criterion present in genetically confirmed hereditary haemorrhagic telangiectasia. *Thorax*. 2022 Jun;77(6):628-630.

5 Topiwala KK, Patel SD, Saver JL, et al. Ischemic Stroke and Pulmonary Arteriovenous Malformations: A Review. *Neurology*. 2022 Feb 1;98(5):188-198.

6 Topiwala K, Patel S, Pervez M, et al. Ischemic Stroke in Patients with Pulmonary ArterioVenous Fistulas. *Stroke* 2021 Jul;52(7):e311-e315

7 Fatania G, Gilson C, Glover A, et al. Uptake and radiological findings of screening cerebral magnetic resonance scans in patients with hereditary haemorrhagic telangiectasia. *Intractable Rare Dis Res.* 2018 Nov;7(4):236-244.

8 Boother EJ, Brownlow S, Tighe HC, et al. Cerebral abscess associated with odontogenic bacteremias, hypoxemia, and iron loading in immunocompetent patients with right-to-left shunting through pulmonary arteriovenous malformations. *Clin Infect Dis.* 2017;65(4):595-603.

9 Kjeldsen AD, Tørring PM, Nissen H, et al. Cerebral abscesses among Danish patients with hereditary haemorrhagic telangiectasia. *Acta Neurol Scand.* 2014 Mar;129(3):192-7.

10 Shovlin C, Bamford K, Sabbà C, et al. Prevention of serious infections in hereditary hemorrhagic telangiectasia: roles for prophylactic antibiotics, the pulmonary capillaries-but not vaccination. *Haematologica.* 2019 Feb;104(2):e85-e86.

11 Clark K, Pyeritz RE, Trerotola SO. Angina pectoris or myocardial infarctions, pulmonary arteriovenous malformations, hereditary haemorrhagic telangiectasia, and paradoxical emboli. *Am J Cardiol.* 2013; 112: 731-4

12 Joyce KE, Onabanjo E, Brownlow S, et al. Whole genome sequences discriminate hereditary hemorrhagic telangiectasia phenotypes by non-HHT deleterious DNA variation. *Blood Adv.* 2022 Jul 12;6(13):3956-3969.

13 Ference BA, Shannon TM, White I, et al Life threatening pulmonary hemorrhage with pulmonary arteriovenous malformations and hereditary hemorrhagic telangiectasia. *Chest.* 1994;106, 1387–1392.

14 Puskas JD, Allen MS, Moncure AC, et al. Pulmonary arteriovenous malformations: Therapeutic options. *Ann Thorac Surg* 1993;56(2):253–258.

15 Kanou T, Shintani Y, Osuga K, et al. Successful lobectomy for central large pulmonary arteriovenous malformation. *Interact Cardiovasc Thorac Surg*. 2012;14:665–7.

16 Reichert M, Kerber S, Alkoudmani I, et al. Management of a solitary pulmonary arteriovenous malformation by video-assisted thoracoscopic surgery and anatomic lingula resection: video and review. *Surg Endosc.* 2016 Apr;30(4):1667-9.

17 Na S-J, Cho HM, Park JS. A case of successful surgical treatment of migraine headaches in a patient with sporadic pulmonary arteriovenous malformations. *J Korean Med Sci* 2009 Apr;24(2):330-2

18 Biçakçioğlu P, Gülhan SŞE, Sayilir E, et al. Surgical treatment of pulmonary arteriovenous malformations. *Turk J Med Sci* 2017 Feb 27;47(1):161-166.

19 Nagano M, Ichinose J, Sasabuchi Y, et al. Surgery versus percutaneous transcatheter embolization for pulmonary arteriovenous malformation: Analysis of a national inpatient database in Japan. *J Thorac Cardiovasc Surg*. 2017 Sep;154(3):1137-1143.

20 Hsu CC, Kwan GN, Evans-Barns H, et al. Embolisation for pulmonary arteriovenous malformation. *Cochrane Database Syst Rev.* 2018 Jan 4;1(1):CD008017.

21 Dutton JA, Jackson JE, Hughes JM, et al. Pulmonary arteriovenous malformations: results of treatment with coil embolization in 53 patients. *AJR Am J Roentgenol* 1995;165(5):1119-25.

22 Gupta P, Mordin C, Curtis J, et al. Pulmonary arteriovenous malformations: effect of embolization on right-to-left shunt, hypoxemia, and exercise tolerance in 66 patients. *AJR Am J Roentgenol* 2002;179(2):347-55.

23 Santhirapala V, Williams LC, Tighe HC, et al. Arterial oxygen content is precisely maintained by graded erythrocytotic responses in settings of high/normal serum iron levels, and predicts exercise capacity. An observational study of hypoxaemic patients with pulmonary arteriovenous malformations. *PLoS One* 2014;9(3):e90777

24 Rizvi A, Macedo P, Babawale L, et al. Hemoglobin is a vital determinant of arterial oxygen content in hypoxemic patients with pulmonary arteriovenous malformations. *Ann Am Thorac Soc* 2017;14(6):903-911.

25 Shovlin CL, Tighe HC, Davies RJ, Gibbs JSR, Jackson JE. Embolisation of pulmonary arteriovenous malformations: no consistent effect on pulmonary artery pressure. *Eur Resp J.* 2008; 32: 162-9

26 Shovlin CL, Jackson JE, Bamford KB, et al. Primary determinants of ischaemic stroke/ brain abscess risks are independent of severity of pulmonary arteriovenous malformations in hereditary haemorrhagic telangiectasia. *Thorax.* 2008; 63: 259-66.

27 Post MC, Thijs V, Schonewille WJ, et al. Embolization of pulmonary arteriovenous malformations and decrease in prevalence of migraine. *Neurology.* 2006;66:202–205.

28 Shovlin CL, Guttmacher AE, Buscarini E, et al, Diagnostic criteria for hereditary hemorrhagic telangiectasia (Rendu-Osler-Weber syndrome). *Am J Med Genet.* 2000 Mar 6;91(1):66-7.

29 Shovlin CL, Simeoni I, Downes K, et al. Mutational and phenotypic characterization of hereditary hemorrhagic telangiectasia. *Blood.* 2020 Oct 22;136(17):1907-1918.

30 Gawecki F, Myers J, Shovlin CL. Veterans Specific Activity Questionnaire (VSAQ): a new and efficient method of assessing exercise capacity in patients with pulmonary arteriovenous malformations. *BMJ Open Respir Res.* 2019 Mar 1;6(1):e000351

31 Shovlin CL, Sodhi V, McCarthy A, et al. Estimates of maternal risks of pregnancy for women with hereditary haemorrhagic telangiectasia (Osler-Weber-Rendu syndrome): suggested approach for obstetric services. *BJOG.* 2008 Aug;115(9):1108-15.

32 Shovlin CL, Gibbs JS, Jackson JE. Management of pulmonary arteriovenous malformations in pulmonary hypertensive patients: a pressure to embolise? *Eur Respir Rev.* 2009 Mar;18(111):4-6.

33 Sapru RP, Hutchison DC, Hall JI. Pulmonary hypertension in patients with pulmonary arteriovenous fistulae. *Br Heart J.* 1969 Sep;31(5):559-69.

34 Le Roux BT, Gibb BH, Wainwright J. Pulmonary arteriovenous fistula with bilharzial pulmonary hypertension. *Br Heart J*. 1970 Jul;32(4):571-4.

35 Muehling BM, Halter GL, Schelzig H, et al. Reduction of postoperative pulmonary complications after lung surgery using a fast track clinical pathway. *Eur J Cardiothorac Surg.* 2008 Jul;34(1):174-80.

36 Schussler O, Alifano M, Dermine H, et al. Postoperative pneumonia after major lung resection. *Am J Respir Crit Care Med*. 2006 May 15;173(10):1161-9.

37 Homma T, Doki Y, Yamamoto Y, et al. Risk factors of neuropathic pain after thoracic surgery. *J Thorac Dis.* 2018 May;10(5):2898-2907.

38 Dualé C, Ouchchane L, Schoeffler P; EDONIS Investigating Group, Dubray C. Neuropathic aspects of persistent postsurgical pain: a French multicenter survey with a 6-month prospective follow-up. *J Pain.* 2014 Jan;15(1):24.e1-24.e20.
